# Centering intersectional breast cancer screening experiences among black, Latina, and white women: a qualitative analysis

**DOI:** 10.3389/fpubh.2024.1470032

**Published:** 2024-11-13

**Authors:** Sienna Ruiz, Kamilah Abdur-Rashid, Rachel L. Mintz, Maggie Britton, Ana A. Baumann, Graham A. Colditz, Ashley J. Housten

**Affiliations:** ^1^Washington University School of Medicine, St. Louis, MO, United States; ^2^The University of Texas, MD Anderson Cancer Center, Houston, TX, United States

**Keywords:** cancer, mammography, patient-centered communication, health equity (MeSH), screening

## Abstract

**Objective:**

Mammography screening guidelines in the United States highlight the importance of informing and involving women when making their breast cancer screening decisions. However, the complexity of interpreting and applying these population-level guidelines can contribute to patient burden. Patient-centered communication strategies can alleviate patient burden, but few consider perspectives from racially and ethnically marginalized populations. We examine diverse women’s perspectives on screening to characterize patient-centered experiences.

**Methods:**

We conducted 28 focus groups with 134 non-Latina Black (*n* = 51), non-Latina White (*n* = 39), and Latina (*n* = 44) participants. We coded participants’ discussion of their screening influences. We used deductive and inductive qualitative methods to identify common themes.

**Results:**

We identified three themes: (1) personal relationships with primary care providers, (2) potential impacts of cancer on families, and (3) interactions with medical systems. Most White participants described trusting physician relationships in contrast to perfunctory, surface-level relationships experienced by many Black participants; high costs of care prevented many Latina participants from accessing care (Theme 1). Diagnosis was a concern for most Black participants as it could burden family and most Latina participants as it could prevent them from maintaining family well-being (Theme 2). While many White participants had general ease in accessing and navigating healthcare, Latina participants were often held back by embarrassment—and Black participants frequently described disrespectful providers, false negatives, and unnecessary pain (Theme 3).

**Conclusion:**

Cultural and structural factors appeared to influence participants’ approaches to breast cancer screening. Structural barriers may counteract culturally salient beliefs, especially among Black and Latina participants. We suggest patient-centered communication interventions be culturally adjusted and paired with structural changes (e.g., policy, insurance coverage, material resources) to reflect women’s nuanced values and intersectional social contexts.

## Introduction

In the United States (U.S.), breast cancer is the most commonly diagnosed cancer and the second leading cause of cancer-related mortality among women (1). Those diagnosed with breast cancer who are members of socially marginalized racial and ethnic groups, are more likely to present with advanced tumors compared to White women (2). Moreover, Black women experience higher breast cancer mortality when compared to women in other racial and ethnic groups (3).

Addressing disparities in breast cancer incidence and mortality is of high priority as reflected in the updated 2024 US Preventive Services Task Force (USPSTF) breast cancer screening recommendations aimed at addressing disparities related to social determinants of health, including racism and low income (4). This updated 2024 USPSTF screening recommendation retains the importance of informing and involving women when making their breast cancer screening decisions (5). To mitigate potential patient burden, patient-centered communication tools designed to clarify patients’ values and preferences can provide strategies to think about healthcare decisions and support well-informed decision making related to breast cancer screening (6). Yet, the few patient-centered communication interventions developed based on the perspectives of racially and ethnically marginalized women have yielded mixed results (7, 8). Furthermore, patient-centered communication interventions increase general screening intention but struggle to alleviate linguistic, cultural, or access barriers to care (8–10).

Women approach breast cancer screening through their experiences of multiple identities—such as gender, race, ethnicity, and socioeconomic status—that must be seen as intersectional rather than as isolated categories (11, 12). When evaluating influences related to mammography among diverse women, researchers often focus solely on race and/or age (13, 14) and overlook how patients’ multiple identities collectively impact their experiences within the US health system. For women who are marginalized based on their race or ethnicity, income, or other identities, it is especially important to understand healthcare in the US through the lens of systemic racism: the institutional reproduction of racist policies and practices (15, 16), and economic inequality, or extreme disparity in economic resources that limit low-income women’s access to care in a fragmented multiple-payer medical system (17, 18).

Systemic racism is reproduced at three multidirectional levels: (1) racial superstructures, or racial ideologies that justify racial inequalities; (2) racial structures, or policies that deny marginalized communities access to social and material resources; and (3) racial substructures, or schemas like emotions or biases that individuals uphold that perpetuate interpersonal discrimination (19). Economic inequality is layered onto systemic racism such that racially and ethnically marginalized communities are more likely to be low-income (20, 21). More research is needed to characterize the intersectional influences related to mammography in the US among women, especially because women of marginalized racial and ethnic backgrounds are more likely to live in segregated neighborhoods (22), lack access to quality insurance (23), be exposed to carcinogens over their lifetimes (24), and be disbelieved in medical encounters (25, 26) than White women. Women may also experience discrimination due to their gender, sexuality, ability, or other marginalized identities. Interventions that do not fully recognize this context can be inapplicable to the real-life obstacles patients experience accessing care. For cancer screening interventions based on patient-centered communication to have a lasting impact, using an intersectional approach to examine the factors that influence women’s mammography cancer screening experiences is needed.

We aimed to characterize the intersectional factors that influence mammography screening decisions. To achieve this aim, we conducted a cross-sectional, qualitative study with Latina, non-Latina Black (hereafter, Black), and non-Latina White (hereafter, White) women participants that examined the experiences of a racially and ethnically diverse sample of women.

## Materials and methods

### Recruitment

The study was approved by the institutional review boards of the University of Texas MD Anderson Cancer Center and Washington University School of Medicine. A detailed description of the recruitment and focus group methods was reported previously (27). Concisely, we recruited women through existing community-engaged partnerships including Project CHURCH (Creating a Higher Understanding of Cancer Research and Community Health; Houston, TX), MACS (Mexican American Cohort Study; Houston, TX), and various community outreach methods (e.g., social media, recruitment flyers; Houston, TX and St. Louis, MO). Focus groups were conducted with self-identified Latina, Black, and White women. Participants met eligibility criteria if they: (1) were between 40 and 75 years of age; (2) were English or Spanish speaking; (3) self-identified as Latina, Black, or White; and (4) self-reported no known increased risk for breast cancer. No known increased risk was defined as: no personal history of breast cancer; no personal history of atypical hyperplasia; no first-degree family member with history of breast cancer; no known underlying genetic mutation; and no self-reported prior thoracic or chest wall radiation therapy.

### Data collection: focus groups

Three trained facilitators led focus groups, ranging from 1 to 3 hours. We offered focus groups in Spanish to those who reported a preference for facilitation language. Focus group guides were pilot tested by the research team in both English and Spanish (27). Before each focus group, participants completed a written informed consent process. After completing the questionnaires and the focus group, participants were compensated with a $50 gift card and a parking or transportation voucher. Recruitment ended when the research team determined similar topics and information were being discussed during the focus groups and the data collected appeared to represent a range of participant experiences (28, 29).

Focus groups were conducted separately by self-identified racial and ethnic background such that participants in each focus group all identified as the same race and ethnicity. They were facilitated using a semi-structured guide (27) based on the Ottawa Decision Support Framework and health behavior theories including reasoned action, planned behavior, the health belief model, and social cognitive theory to address personal and environmental characteristics of the decision processes, and participants’ decisional needs, outcomes, and support (30–37). For this analysis, the research team focused on two questions:

*Question 1: What are some of the things that you might consider or think about when deciding whether to obtain a mammogram? What about when deciding how often to obtain a mammogram?*

*Question 2: Who or what might influence your decision to obtain a mammogram? Probe: Healthcare providers Family? Friends? Websites? Other reading material?*

We focused our analysis on the overlapping and co-constitutive influences produced during the robust focus group discussions (i.e., participants discussed diverse topics producing textured, rich, and thick data). Participants’ rich discussions in relation to these questions prompted our analysis into the intersections of participants’ identities and social contexts informing their thoughts about breast cancer screening decisions.

A professional transcription service transcribed all focus group recordings verbatim and translated Spanish transcripts into English. Spanish transcripts were reviewed by a minimum of one native Spanish speaker (27). Using NVivo 11, transcripts were coded independently by a research assistant and study PI (KAR and AH), and discrepancies were resolved with a third investigator (GC). Based on the responses to the two questions of interest, we determined that responses among Latina participants were similar regardless of language preferences. Therefore, we discuss Latina responses collectively for analytical purposes.

### Level one analysis (Anderson model)

Our first level of analysis used deductive coding based on the Andersen Behavioral Model of Health Services Use to categorize the topics participants named as screening influences (38). This model outlines potential facilitators and barriers for future health interventions and describes three main factors that influence individuals when considering healthcare services: predisposing, enabling, and need factors (38). Predisposing factors refer to pre-existing characteristics, such as demographic details or health beliefs (e.g., susceptibility, risk) that can influence an individual’s decision to use healthcare services (38, 39). Enabling factors include resources facilitating healthcare access (39). Need factors include an individual’s perceived and evaluated general state of health and symptoms (40). These factors established the initial deductive codes used to conduct a qualitative descriptive analysis of the data (Table 1) (41–43).

**Table 1:** Influencing factors for breast cancer screening (38–40).

Predisposing factors	Age, race, knowledge, beliefs, mistrust, religiosity, fears and fatalism, lifestyle and environment
Enabling factors	Health insurance, access to care, health utilization, customer service
Need factors	Physician recommendation, family/personal history, pain or discomfort, family responsibilities

### Level two analysis (thematic)

A second-level qualitative thematic analysis was conducted to deepen the examination of intersectional aspects (e.g., race, ethnicity, sociodemographic characteristics) of participants’ mammography experiences—beyond what the Andersen model could examine. Specifically, this qualitative thematic analysis was done by combining the level-one deductive approach using the Anderson model with inductive coding, wherein codes were identified directly from the data (44). Inductive coding allowed the research team to derive themes that identified the relational, rather than categorical, ways in which participants discussed making screening decisions across the dataset (45, 46). After second-level qualitative thematic analysis, research team members discussed and identified which themes were most salient across all participant groups (i.e., most transcripts included content related to the theme) and was representative of participant discussion (i.e., most participants in a focus group talked about topics related to the theme) (47). Team members reviewed themes across groups to examine variability by participants’ race, ethnicity, and sociodemographic characteristics and their experience of a theme as identified within the dataset.

## Results

A total of 134 people participated in 28 focus groups. Over a third (38%) of the participants were Black (*n* = 51), 33% were Latina (*n* = 44), and 29% were White (*n* = 39). Most Latina participants (60%) were Spanish-speaking (*n* = 26). About half of the participants (48%, *n* = 64) received Medicare, Medicaid, or Medical Assistance, while 62% (*n* = 83) reported having private or group insurance (participants could report multiple forms of insurance). Most participants (84%, *n* = 113) reported having a primary care physician (PCP) compared with 15% (*n* = 20) who did not. All participants self-identified as women (Table 2).

**Table 2:** Sociodemographic characteristics.

Race/Ethnicity	Black (*N* = 51)	White (*N* = 39)	Latina–English (*N* = 18)	Latina-Spanish (*N* = 26)	Total (*N* = 134)
	M (SD) Range	M (SD) Range	M (SD) Range	M (SD) Range	M (SD) Range
Age (years)	54.80 (10.32) 40–73	61.45 (11.38) 40–75	54.06 (10.43) 40–70	52.04 (8.84) 40–73	56.10 (10.88) 40–75
Education (years)	14.61 (1.86) 12–18	15.18 (1.52) 12–18	13.56 (2.45) 6–17	9.15 (3.63) 1–14	13.57 (3.19) 1–18
	Median [Range]	Median Range	Median Range	Median Range	Median Range
Income	$30,000–$40,000 [<$10,000- ≥ $100,000]	$60,000–$70,000 [$20,000- ≥ $100,000]	$50,000–$60,000 [<$10,000- ≥ $100,000]	$20,000–$30,000 [<$10,000–$70,000]	$40,000–$50,000 [<$10,000- ≥ $100,000]
	N (%)	N (%)	N (%)	N (%)	N (%)
Employment Status					
Employed	25 (49.0)	19 (48.7)	11 (61.1)	5 (19.2)	60 (44.8)
Unemployed	4 (7.8)	2 (5.1)	1 (5.6)	0 (0.0)	7 (5.2)
Homemaker	1 (2.0)	2 (5.1)	1 (5.6)	18 (69.2)	22 (16.4)
Retired	15 (29.4)	16 (41.0)	2 (11.1)	3 (11.5)	36 (26.9)
Other	6 (11.8)	0 (0.0)	3 (16.7)	0 (0.0)	9 (6.7)
Government Insurance (e.g., Medicaid, Medicare)					
Yes	21 (41.2)	21 (53.9)	8 (44.4)	14 (53.9)	64 (47.8)
No	30 (58.8)	16 (41.0)	10 (55.6)	12 (46.2)	68 (50.8)
Unsure	0 (0.0)	1 (2.6)	0 (0.0)	0 (0.0)	1 (0.8)
Private or Group Insurance					
Yes	35 (68.6)	31 (79.5)	10 (55.6)	7 (26.9)	83 (61.9)
No	15 (29.4)	7 (18.0)	7 (38.9)	18 (69.2)	47 (35.1)
Unsure	1 (2.0)	0 (0.0)	1 (5.6)	1 (3.9)	3 (2.2)
Primary Care Physician					
Yes	43 (84.3)	37 (94.9)	16 (88.9)	17 (65.4)	113 (84.3)
No	8 (15.7)	1 (2.6)	2 (11.1)	9 (34.6)	20 (14.9)
Mammogram History					
Yes	47 (92.2)	37 (94.9)	17 (94.4)	18 (69.2)	119 (88.8)
No	4 (7.8)	2 (5.1)	1 (5.6)	7 (26.9)	14 (10.5)
Unsure	0 (0.0)	0 (0.0)	0 (0.0)	1 (3.9)	1 (0.8)

Percentages may not total 100.0 due to rounding and/or missing data. Age is the age reported when participants were screened for eligibility. Education is reported in approximate years (e.g., high school graduate = 12 years). *N* = 13 refused to report income. Employment “Other” category includes unable to work/disabled, student, and other. Mammogram history is whether women ever had a breast cancer screening mammogram. Another version of this table has been previously published in Housten et al. (27).

We identified three themes: (1) Personal relationships with PCPs, (2) Potential impacts of cancer on patients’ families, and (3) Interactions with medical systems. Presentation of each theme varied the most by participants’ racial or ethnic identities, and therefore we describe how the theme varied using racial and ethnic groups. In parentheses, we provide participant sociodemographic characteristics like age, income, and insurance status. Annual household income levels were defined as: lower <$50,000; higher ≥$50,000. All participant quotes are attributed to pseudonyms. For additional exemplar quotes, see Table 3.

1. Personal relationships with primary care providers

**Table 3:** Results summary.

Theme	Andersen codes	Participant group		
		Black participants	Latina participants	White participants
Personal Relationships with PCP	Cost-covering services (Enabling) Physician recommendation (Need)	Perfunctory relationships, few personalized recommendations “I just listen to my PCP or my OB-GYN whenever they write the script then, I just go. And it’s almost every year.” (Cynthia, 44 years, higher income, private insurance)	Absence of PCP due to high costs of care “I have never had the screening…because the insurance I have does not cover a lot—the deductible is very high. But I need to find out where there’s a place that is not so expensive.. I am not working at the moment…a lot of times we have the money to do other things and the most important thing, which is health, we leave that for later.” (Camila, 41 years, lower income, public and private insurance)	Close, trusting relationships with PCPs “I go to my internist once a year. He tells me what I have to have done. He tells me when it’s time for a mammogram…he has been following me for twenty-five years, and I trust him. And he tells me what to do and I listen.” (Megan, 74 years, lower income, public insurance)
Potential Impacts of Cancer on Families	Family history of cancer (Need) Family responsibilities (Need)	Worries about burdening family members if they got cancer “I am very independent, so I am keeping [screening] early, losing less time that I have to depend on someone, because if…way, way down the road…need to depend on them…the worse off you are.” (Sabrina, 69 years, lower income, public & private insurance)	Saw cancer as a threat to their roles maintaining family well-being “I do not want to break my children. I have a son and daughter, and I always think about I do not want to be a burden to them. I have to take care of myself’.” (Linda, 71 years, higher income, public insurance)	Motivated to get screened due to family experience with cancer “My mother…had breast cancer twice. And I have a really good friend going through breast cancer treatment right now…So that definitely influences me to do it yearly.” (Christina, 65 years, higher income, private insurance)
Interactions with Medical Systems	Fears and fatalism (Predisposing) Beliefs in health-seeking behavior, physician roles (Predisposing) Customer service (Enabling)	Experiences of pain, false negatives, or disrespectful providers that could inspire medical mistrust “And I do not understand why women have to go through so much devastation and pain when we have things wrong with us…I’m thinking it’s just in the big business and interest of the pharmaceutical company that we still have these problems with the research…But they are coming up with more cancers…and then [I] have to keep coming back and get cut up.” (Sylvia, 71 years, lower income, public & private insurance)	Embarrassment around breast cancer, complicated beliefs surrounding health-seeking behavior “Well, sometimes you feel embarrassed about going to the doctor, right?…It happened to me when the doctor checked me out…we are not used to being touched by other men. That’s why many people do not go to the doctor, because they feel embarrassed.” (Sandra, 45 years, lower income, public insurance)	Had customer service-like experiences of and expectations for care “It took me 15 min. It was the easiest appointment I had ever been to in my life. The girls were friendly. It was like I had coffee. I felt like I was at a spa…I did it right here…and it was 15 min, so I could find the time.” (Natalie, 42 years, no reported income, private insurance)

Name are pseudonyms; Annual household income levels were defined as: lower < $50,000; higher ≥ $50,000.

Most Black participants described perfunctory, routine relationships with their PCPs, where providers gave consistent reminders and general recommendations for screenings. For example, Gayle (58 years, higher income, private insurance) described how she decided to get screened in the context of a more surface-level relationship with her PCP:

“When you go see your primary care physician [for] blood work, mammograms… and they look at your chart and they are like, ‘You have not had your [screening] this year’. The doctor suggests you should have it, then you do it.”

Thus, physician recommendations helped many Black participants create routines for screening but did not necessarily support patient-centered communication. Tessa (49 years, Black, lower income, private insurance) described how general physician prompts got her to schedule screenings:

“[The doctor says] ‘It’s time for your mammogram,’ and I’m like, ‘Yes, ma’am’…But I do not just like, [say] ‘Oh, I’m just going to schedule my mammogram.’”

In contrast, some Black participants, described closer relationships with their PCP with whom they could discuss screening to determine initiation and frequency of screening. For example, Gabriella (45 years, did not report income, public insurance), cited her positive experience with her PCP as a motivator for screening:

“I had the most incredible primary care physician. She is so empathetic. She is an active listener, and I really feel as though she is my partner in my quest for just a good health-related [quality] of life…And when I told her that I had [family] history [of breast cancer], she said…‘I’ll go and get you approved for a mammogram and then we are just going to get this done.’”

Many Latina participants shared that they did not have a PCP because of high cost of care, inadequate insurance, or a lack of any insurance. Dani (68 years, lower income, public insurance) described the difficulties she experienced in finding accessible, low-cost screening options without a physician:

“I have not had [a mammogram] in a long time, and I do not have a primary doctor…So, I went to [a local low-cost clinic] and they wanted a hundred and something…So, I did not do it, and…three or four years passed… [Now] I’ve funding, so I have one done about two months ago…To me that was a blessing…Right now, all I have is my social security check. And…that is for bills, and sometimes you do not have anything left, not even to buy food …Having utility bills is more important to me than a mammogram…I try to look for places [where]…you can get your mammogram free.”

Limited contact with providers and experiences with high costs of care appeared to deter many Latina participants from screening. Betty (59 years, lower income, no insurance) expressed that even though she would have liked to continue screening, loss of insurance kept her from doing so:

“When I had my insurance, I would even go every six months, because the doctor told me, ‘Come in six months.’ And I went. But now I no longer have an insurance, nothing…I have not really sought for a place where they charge less, because they do charge me $400, $500 dollars. It’s a lot of money. And now my husband is out of work too…but I do not want to abandon [screening].”

Some Latina participants like Yessenia, cited insurance coverage and lower costs as extremely helpful facilitators for getting screened, as insurance meant they could get a provider’s referral for a mammogram. Yessenia (58 years, higher income, public insurance) said:

“I think for me [my biggest influence] originally was cost. Of course, once I got insurance then…it’s already got into my insurance plan. I do not have to pay for it. Well, of course, I’m going to do it.”

In contrast to the common experience among Black and Latina participants’ surface-level or perfunctory relationships with physicians, or struggles with access to screening because of difficulties with insurance or finances, many White participants described a more engaged relationship with physicians, resulting in trusted advice about breast cancer screening. Among participants, a greater proportion of White participants reported insurance coverage and a PCP than Black and Latina participants. Liz (69 years, higher income, public and private insurance) detailed how recommendations from a physician who knew her medical history inspired her to get screened:

“And so [my physician] can recommend to me [when to get screened], and I think that is one of the benefits of having a primary doctor you can trust who will keep up with how active you are, what you are doing, who can really decide [whether] you need to keep doing this. And so I would trust [my doctor] because she knows my lifestyle, she knows what I am doing.”

Similarly, Elisa (58 years, higher income, public and private insurance) discussed how the gynecologist she has seen for years shaped her approach to screening:

“I’ve had a long-term gynecologist, and from the beginning she said it’s best to have a mammogram every year. So, I thought she was in the best position to give me the best advice.”

Unlike other White participants, some mentioned their experience with clinical constraints (e.g., limited appointment times that hindered their screening decisions). Erin, (68 years, middle income, public and private insurance) shared that she needed to seek out screening information on her own because she did not find her discussions with providers sufficient because of their lack of time:

“I think it’s really important that we are educated. And how much [are the] PCPs…educating you? …If they do not have time, one of the things that you…can get to is learn more about mammogram screening.”

2. Potential impacts of cancer on families

Often, Black participants described the potential burden of a cancer diagnosis on their families and how screening could ease their worry about the possibility of needing to rely on others if they had cancer. This was particularly relevant to many older Black participants, who saw screening as a way to maintain their autonomy and independence by increasing their health awareness. Maria (74 years, lower income, public and private insurance) said that she used screening to avoid relying on her adult child for care and as a way to express self-love:

“I only have one son, and I do not want to have to depend on him or his wife. I am divorced, and I do not want to depend on anyone…I love me, and I am going to try to take care of me. If they tell me I need to come every three months, I’d go every three months. If [doctors] were telling me, I do not need to see you, but once a year, then I go once a year. Whenever they tell me to come, I come.”

For some younger Black participants, screening was a way to “take charge” of their health in light of the multiple forms of discrimination they faced as Black women. Tina (44 years, higher income, private insurance) remarked:

“There’s nothing for me to think about [when it comes to screening].. when it comes to my health, it’s life. I have children …I think healthcare is just so important, and as a Black female, I think we have a lot of that we are up against when it comes to health. And it’s just important to me like, really a priority.

A few Black participants, however, had families who inspired them not to screen. Hazel (75 years, lower income, private insurance) discussed her “personal choice” to avoid exams because she felt like her family members and friends over-relied on medical care. She said:

“I have a sister who has a doctor for everything… I truly can say that some people overdo it. I have friends who just obsess…I’m totally the opposite…I try to use a level head with it…It’s a point where you have to use common sense. And if [providers] keep looking, they are going to find something.”

Many Latina participants described screening as a way to take care of their health and ensure they could care for their families in the long term. Lower-income Latina participants who lacked insurance believed familial health depended on maternal health and that screening could help them avoid potential death from breast cancer that would imperil the family. Valeria (44 years, lower income, no insurance) said:

“We’re the engine of the family and we have to take care of our children…if they do not have their mother, if we are not in the family, who’s going to protect them? On the contrary, this is when the family is paralyzed. And so we are okay for our children to be okay.”

Another lower-income participant, Ana (43 years, lower income, no insurance), discussed how screenings could help reassure her and her family of her well-being:

“Other things that influenced me personally quite a lot to have those exams are my children, my family, because I think that if you are fine your children are going to be fine.”

Some lower-income Latina participants, like Camila, also cited the importance of prioritizing the family as a reason for why they did not get screened, contextualizing this importance within their positions as immigrants who needed to continuously provide for their families. Camila (41 years, lower income, public and private insurance) said:

“In fact, we are immigrants…a lot of times we do not take care of our health in order to send money to our family who is in our country, and we forget about ourselves.”

White participants were often influenced to screen based on watching a family member or a friend deal with a cancer diagnosis or from screening guidance from friends and family based on their experiences. For example, Erin (68 years, higher income, public and private insurance) described how her mother’s experience with lung cancer pushed her to screen:

“I will say one of the reasons I did not cancel [my mammogram] was my mom had a lung cancer diagnosis last year, and it was a very early detection…I knew plenty of people affected by cancer but it had never hit my family…I had waited [to get screened until] I was forty-two… had my mom not had this cancer diagnosis, I probably would have found a reason to cancel that appointment.”

Loved ones’ experiences with cancer could influence White participants toward screening to also avoid later detection of cancer and the need for more intensive cancer treatments. Julia (77 years, lower income, public and private insurance) mentioned that a friend of hers pushed her to screen:

“I have a friend who is older than I am…and her friend…went to get a mammogram…she was already way past the seventy-five…and they found something, and she had to have treatment. So she’s telling everybody…‘Do not let them tell you to stop [screening]. I never had anything before, and I’ve had it now’…I think she’s been an influence, too.”

A few White participants described relationships with family and friends that could dissuade them from screening. For example, Margot (74 years, higher income, public and private insurance) said that an acquaintance shared advice related to screenings that led her to avoid them:

“[A] person…told me I’m old enough to probably not have to worry about [screening], there’s no family history. And I know that’s not always an indication, but I just thought, well, that’s okay, one less thing that you have to worry about. So, I have not gone now for a couple of years.”

3. Interactions with medical systems

Many Black participants described experiences in which medical providers failed to explain the mammography process, were uncompassionate about delivering diagnostic results, or caused participants unnecessary physical pain. This pattern could contribute to feelings of mistrust in medical systems for Black participants, especially those who had lower incomes. Experiencing mistreatment and dehumanization in medical settings appeared to contribute to many Black participants’ skepticism of screenings. Ramona (69 years, lower income, public and private insurance) recounted her last mammogram and shared her difficult experience:

“They called me, the nurse called me on the phone…. ‘You need to come in, and we are going to do a biopsy’…you are not putting fear in my heart because I do not have to go through this…And then they call you like you are some kind of piece of meat. They’re going to throw you in, throw you out…they are going to find something because you got to die with something.. It’s up to you to make a decision, whether you are going to let them slice you, bog you down…degrade you in a way. But I am not going to let them do that… I mean, doctors…have to get paid. You know what I’m saying? So y’all got to find something. But it’s not going to work with me.”

Jessica (69 years, lower income, private insurance), talked about how after caring for an aunt with breast cancer, she had to deal with the scare of an abnormal mammography result. She expressed how, at the time, she felt that providers were inconsiderate toward her, impacting her view on screening:

“When I went last year the doctor, I felt, was rude…He called me and said, ‘You need to come in.’ So I did. He pulls out this fax and says ‘Okay. When you had your X-ray done…’ First of all…I had to wait a couple of days so I could get into your office. Then, I get there, and you show me a fax that came in. To me, you should have had the records…to come over and show me the X-ray, so I could have an idea of what I was going to have to face. So in the meantime, it’s going to cost $5,000 to get this done. I sat there and I had to think about that situation because I just experienced that in our family for the first time, of having a female in our family to survive cancer. So that was very scary.”

Some Black participants, however, described positive experiences with providers and screenings that motivated them to continue to get screened. Rosie (72 years, lower income, public insurance) described her positive experiences:

“And I have not had any painful experiences..[providers] let you know what they are going to do ahead of time, ‘Okay, we are going to position this. It may be a little bit tight, but just let me know if there’s any discomfort.’ So they really explained and they were very caring…that’s what moves me to get it done.”

Latina participants appeared to express beliefs that people may seek medical care only when health problems and symptoms arise and that the topic of breast cancer was deeply embarrassing for women to discuss with providers. These beliefs about health-seeking behaviors and embarrassment surrounding breast cancer could impact how Latina participants approach routine medical care, such as breast cancer screening. For example, Evelyn (46 years, higher income, private insurance), discussed how she only got screenings because she worked near a clinic, and that otherwise she avoids medical care:

“[Screening] is convenient for me. I think that if I did not work so close to the clinic, I probably would not go yearly…I try not to go to the doctor. That’s just my personal preference. I mean if I get sick, I’m not running to go to the doctor. I’ve never been like that. I’d have to be like dying to go to the doctor or I need stitches or something, but that’s just me.”

Veronica (49 years, lower income, no insurance), discussed how symptoms pushed her to get screened, but that she had to deal with embarrassment during the screening process:

“Sometimes it is more than a year…Because not till I go…I felt a little lump on my right breast, and I went to a doctor. It’s embarrassing for me to show it, but she sent me for another mammogram and the doctor said it was just like a lump of fat, not a tumor…[but] to me it’s very, very embarrassing to show the breast to a doctor or…I mean, it’s a bad thing.”

Not all Latina participants expressed a belief that women may be embarrassed about screening or that people may only seek care after feeling symptoms. Some, like Ana (43 years, lower income, Latina, no insurance) aligned their beliefs in women’s strength with the act of seeking screening:

“The woman…stick[s] with…pain, it’s as if she makes it part of her life. So I think that being informed opens your eyes and makes us have those examinations, the breast cancer screening and other examinations for women.”

In contrast to Black and Latina participants, many White participants perceived a general ease of accessing and navigating health systems presenting themselves as patient consumers (48) who regarded healthcare treatments as market services to procure. If screenings did not meet participants’ standards, they spoke of their ability to switch providers, facilities, or screening type as part of their power as patient consumers. Reyna (43 years, higher income, private insurance) talked about how if a provider was not meeting her expectations, she could replace that provider:

“Most of us are educated to a point where we know how to…clear out information that’s not needed and we depend on our doctors for that. But if that relationship is not working properly, then you go outside until you find something that satisfies your questions and your fear.”

Similarly, Sophie (73 years, lower income, public and private insurance) detailed how, when her provider did not get the test results she expected, she was able to request the follow-up care that she wanted:

“I had gone into the gynecologist. The gynecologist could not find anything, and I told her that…there is something wrong in my left breast. And I said, ‘When you write up the orders, I want it as diagnostic because if they do not put it as diagnostic, you do not get an ultrasound.’ So…I went into the ultrasound, and it found something…gynecologists do not always know…gynecologist[s] can make mistakes.”

Even though many White participants discussed their processes of accessing higher quality care, some mentioned difficulties navigating health systems that could not be solved by seeing different providers. Erica (43 years, higher income, private insurance) talked about challenges finding childcare or time for the screening that a customer service approach would not be able to address:

“[Getting screened is] hard, too, because…you know, I have to take care of me…But in the whole scheme of things and daily grind, I have two little kids, I’ve got a five-year old and a three-year old, and they come first, and then there’s me…and that just happens to you when you are a mom, and it’s hard.”

## Discussion

In our study, racially and ethnically diverse participants described how their relationships with providers, the possible impact of breast cancer on their families, and interactions with medical systems impacted their screening experiences. This revealed how participants’ multiple identities appear to collectively impact their experiences within the US health system, including their beliefs surrounding familial responsibility, independence, health-seeking behavior, and embarrassment around breast health and structural contexts (e.g., economic and insurance status, systemic racism replicated in the clinical encounter) that are inextricably linked to the screening approaches women take. While influential factors for mammography like familial roles (49), physician advice (13), and embarrassment (50) were previously identified among Black, White, and Latina women respectively, we found that participants’ varied social contexts impacted their screening in complicated ways. Salient cultural concepts among Black and Latina communities (51, 52), such as beliefs in independence or familism, may have contributed to screening motivations, but these intentions could be challenged by cost and insurance barriers, discrimination in medical contexts, or differing beliefs surrounding feminine modesty. Beliefs in independence or familism could even dissuade participants from screening if, for example, providing for family members after immigrating to the US took precedence over engaging in preventive health measures or if comparing one’s screening experience to a family member’s precluded screening.

Women’s identities within social contexts vary, and a participant who experiences racial or ethnic marginalization may also have a close relationship with a provider that enables robust patient-centered communication about breast cancer screening. Therefore, women approach mammography in a multidimensional way. Systemic racism at the structural level, in the form of limited access to medical care among racially and ethnically marginalized populations due to high costs of care, lack of adequate insurance coverage, and geographic and social distance between patients and providers, can also impact provider schemas about women of color, perpetuating medical mistreatment that causes medical mistrust and deters screening. White participants’ experiences may have been impacted by greater financial resources to select insurance and providers. This involves intersections between interpersonal familial, acquaintance, and provider relationships, structural barriers to care, and potentially contradictory beliefs surrounding screening.

Our findings suggest patient-centered communication breast cancer screening interventions should address both the individual nuance in approaches to breast cancer screening and the broader social contexts in which screening decisions occur to advance equity. Currently, the Community Services Preventive Task Force (CSPTF) endorses multicomponent interventions that increase community demand for and access to screenings as well as increase provider delivery of screening services (53). Patient-centered communication interventions can increase relevance of interventions to patients by including discussion prompts for women and their families or providers that validate culturally-motivated beliefs related to embarrassment or autonomy (54), role-playing activities for patients and providers to improve patient activation of mammography decisions (54, 55), or cultural and structural competency training for providers to raise awareness of and responsiveness to potential patient mistrust or structural barriers to care (56). Many participants in this study discussed cost and insurance barriers. While the Affordable Care Act (ACA) covers financial costs for preventive services, including breast cancer screening, there may be a lack of patient awareness which could be an opportunity for patient-centered communication. Ultimately, patient-centered communication interventions can help better inform patients on mammography; yet, they may not address the systemic issues highlighted in this study and others, such as patients’ strained relationships with providers or the economic and policy issues that may impair their access to and quality of care, even when financial costs associated with cancer screening are covered by health insurance. Multicomponent interventions that address policy (e.g., expansion of public insurance, paid leave from work for screenings) and services offered by medical institutions (e.g., cost-covering programs, financial navigators) in tandem with patient-centered interventions are crucial for decreasing cancer disparities.

### Clinical implications

Based on our findings, we suggest that researchers and clinicians can directly address patients’ cultural and structural screening influences when designing and implementing screening programs. Clinicians can consider integrating patients’ personal narratives that speak to cultural beliefs surrounding mammography, educational information on relevant national and state policies for mammography coverage for insured participants, and resources for low- and no-cost screening opportunities accessible to citizens and non-citizens into patient-centered communication interventions. This may include language-appropriate and culturally-responsive motivational interviews on breast cancer screening (57), educational workshops conducted at relevant community locations like churches and led by community health workers (CHWs) on screening benefits under the Affordable Care Act (58), and brochures and group discussions on payment resources (59). Such educational interventions improved screening uptake and knowledge among patients (60). In addition, clinicians can also touch upon potential patient mistrust by training providers and staff members in trauma-informed approaches to care that include providing an overview of what will happen during the appointment, allowing patients to make choices about non-hindering elements of the appointment, and affirming patients’ potential anxieties during the appointment (61). Trauma-informed care can increase patients’ sense of control and trust during medical procedures and feel more positive about their experiences with providers (62). Within institutions, clinics can consider addressing medical mistrust by engaging CHWs or patient navigators who can be members of the patient community, speak the language of those in the community, and interact with patients outside of medical settings to educate on and assist with screening (63). Institutions can also work to provide patients with more resources like mammography vans, cost-covering services, or outreach programs that improve the overall accessibility of screening. To aid in efforts to improve mammography accessibility at the societal level, clinicians can engage in local political advocacy through interdisciplinary collaboration (64) that endorses improved financial support, living conditions, access to low-cost healthcare resources, public investment in educational and social services, and environmental measures to intervene upon known social determinants of health for low-income, racial/ethnic minority populations (65). Clinicians can also advocate for the expansion of public insurance at the state and national level by collaborating with interest groups or external organizations, increasing public awareness of the issue through public scholarship and media engagement, and facilitating inter-institutional partnerships as a means of applying their medical expertise to larger political conversations (66).

### Study limitations

Participants’ self-report of their sociodemographics could have neglected key aspects of their identities that may have revealed within racial and ethnic group differences. We could have asked for further contextual aspects of their identities; however, we did not want to participants to feel additional burden or exposure by sharing immigration status or other aspects of their identities that may exacerbate a tenuous circumstance. Furthermore, we did not ask participants about the household number and thus do not have a complete understanding of participants’ family finances. Participants were recruited from two regions in the United States and were ages 40–75 years. Our study provides a rich analysis of their experiences. Future work examining experiences across various geographic regions and younger or older ages could further our understanding of breast cancer screening influences. Finally, this study was conducted with the research team’s perspective of the participants as cisgender women. Future studies examining people of other genders’ perspectives on breast cancer screening will also be important for the field.

## Conclusion

Three main themes appeared to influence participants’ approach to breast cancer screening: personal relationships with primary care providers, potential impact of cancer on families, and interactions with medical systems. These themes were expressed differently by participants of different racial and ethnic groups. Most Black and Latina participants expressed cultural motivators or detractors to screen in the face of structural barriers to care. In contrast, White participants often discussed their ease of navigating care and negotiation for improved health services. Future interventions should acknowledge and address these factors to reflect the health needs of diverse patients who undergo breast cancer screening while also attending to the intersectional nature of screening barriers.

## Data Availability

The datasets presented in this article are not readily available because of the qualitative nature of this dataset. Requests to access the datasets should be directed to AH, ahousten@wustl.edu.
